# Potential damaging mutation in *LRP5* from genome sequencing of the first reported chimpanzee with the Chiari malformation

**DOI:** 10.1038/s41598-017-15544-w

**Published:** 2017-11-09

**Authors:** Manuel Solis-Moruno, Marc de Manuel, Jessica Hernandez-Rodriguez, Claudia Fontsere, Alba Gomara-Castaño, Cristina Valsera-Naranjo, Dietmar Crailsheim, Arcadi Navarro, Miquel Llorente, Laura Riera, Olga Feliu-Olleta, Tomas Marques-Bonet

**Affiliations:** 1 Institut de Biologia Evolutiva (CSIC-UPF), Departament de Ciències Experimentals i de la Salut, Universitat Pompeu Fabra, Doctor Aiguader 88, Barcelona, 08003 Spain; 2 Fundació Mona, Carretera C-25, s/n, Riudellots de la Selva, 17457 Girona Spain; 3 Catalan Institution of Research and Advanced Studies (ICREA), Passeig de Lluís Companys, 23, Barcelona, 08010 Spain; 4 CNAG-CRG, Centre for Genomic Regulation, Barcelona Institute of Science and Technology (BIST), Baldiri i Reixac 4, Barcelona, 08028 Spain

## Abstract

The genus *Pan* is the closest related to humans (*Homo sapiens*) and it includes two species: *Pan troglodytes* (chimpanzees) and *Pan paniscus* (bonobos). Different characteristics, some of biomedical aspect, separate them from us. For instance, some common human medical conditions are rare in chimpanzees (menopause, Alzheimer disease) although it is unclear to which extent longevity plays an active role in these differences. However, both humans and chimpanzees present similar pathologies, thus, understanding traits in chimpanzees can help unravel the molecular basis of human conditions. Here, we sequenced the genome of Nico, a central chimpanzee diagnosed with a particular biomedical condition, the Chiari malformation. We performed a variant calling analysis comparing his genome to 25 whole genomes from healthy individuals (bonobos and chimpanzees), and after predicting the effects of the genetic variants, we looked for genes within the OMIM database. We found a novel, private, predicted as damaging mutation in Nico in *LRP5*, a gene related to bone density alteration pathologies, and we suggest a link between this mutation and his Chiari malformation as previously shown in humans. Our results reinforce the idea that a comparison between humans and chimpanzees can be established in this genetic frame of common diseases.

## Introduction

The genus *Pan* is the closest related to humans and it includes two species: *Pan troglodytes* (chimpanzees) and *Pan paniscus* (bonobos). The former is comprised of four subspecies, commonly named after the geographical location they inhabit within Africa: *Pan troglodytes ellioti*, also known as Nigeria-Cameroon chimpanzees, *Pan troglodytes troglodytes* or central chimpanzees, *Pan troglodytes verus* or western chimpanzees and *Pan troglodytes schweinfurthii* or eastern chimpanzees. Little is known about bonobo population substructure. Divergence between our species and the genus *Pan* is estimated to have occurred between 5 to 7 million years (Myr) ago^1^.

Due to the great genetic similarities, chimpanzees and bonobos have classically been proposed as animal models to study human diseases of several natures^2–4^. Nevertheless, it is also obvious that there are differences between our species and our closest living relatives. Some of the most common medical conditions in humans are rare in chimpanzees, such as menopause, Alzheimer disease, HIV progression to AIDS or carcinomas^5^. A great number of the biomedical differences between us and not only chimpanzees and bonobos but all of the non-human primates, can probably be explained by lineage specific distinctive features. One of the best examples are back disorders caused by our bipedal posture^6^.

Apart from the evident differences, it is unquestionable that the similarities can be used in our favour to acquire novel knowledge of our own species. In fact, the availability of the chimpanzee genome has been useful to answer several relevant questions about the evolution of the human genome and, thus, can be helpful to understand how certain changes have been important to human health^7^. A previous publication analysed the genome of a chimpanzee with an analogous disease to humans, the Smith-Magenis syndrome^8^, showing that both species do share the same gene deletions.

The Chiari malformation type 1 or, simply, Chiari malformation, is a condition described in 1891 by the Austrian pathologist Hans Chiari^9^. It is characterized by the shift of the cerebellar tonsils into the spinal canal. The displacement is produced through the *foramen magnum* and it must be 5 mm or more to be considered as Chiari malformation^10^. This trait is frequently associated with syringomyelia^11,12^. The most common symptoms are headaches, ocular disturbances such as retro-orbital pressure or pain and blurred vision and otoneurological disturbances like disequilibrium and pressure in the ears^13^.

Hans Chiari described a total of three malformations known as the Chiari malformations types 1–3. To date, the list has been extended with at least another three (reviewed by Poretti *et al*.^14^). They all are very heterogeneous entities, even within the same type.

There is not a clear and straightforward genetic determination for these conditions. Previous studies suggest a genetic background for them since many familial cases have been reported^15–18^. In fact, the first clinical trial to accomplish a genetic analysis of this malformation has begun and it is currently recruiting patients (ClinicalTrials.gov Identifier: NCT00004738). Some genetic disorders have already been connected to the Chiari malformation, most of them directly or indirectly related to bone density impairment or bone malformation, such as achondroplasia, hypophosphatemic rickets, familial osteosclerosis or Paget’s disease of the skull^19^.

Here, we studied the complete genomic sequence of a chimpanzee with Chiari malformation to understand the extent of similarities with human patients and their purported molecular profiles.

## Materials and Methods

### Individual

**Figure 1:**
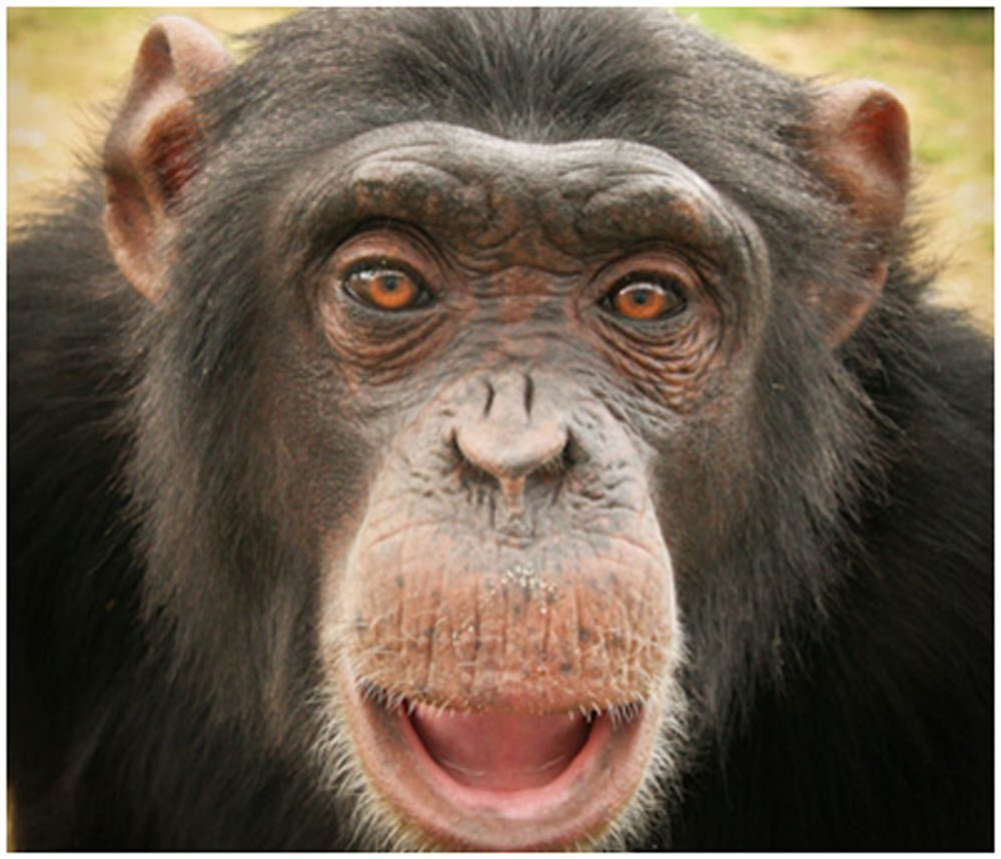
Nico.

Nico (Fig. 1) was born in captivity from two wild-born parents.

Nico presented serious psychological problems that made him injure himself to the point of losing his left hand. He also presented some physical symptoms like alterations in his jaw. For diagnosis purposes, he underwent magnetic resonance imaging (MRI). This is how we discovered his Chiari malformation and the associated syringomyelia. To improve his state, he was successfully operated to decompress the affected area.

Here, we report Nico as the first chimpanzee diagnosed with the Chiari malformation.

### Dataset

We followed TruSeq DNA PCR-Free Library Preparation protocol to obtain a final library insert size of 350 bp from the peripheral blood of Nico, a male chimpanzee (*Pan troglodytes*). The blood was obtained in a routine veterinary check at Fundació Mona following ethical rules. Then, his whole genome was sequenced to a mean coverage of 30X on an Illumina HiSeq X Ten sequencing platform with 150 bp paired-end reads.

Complementary to this genome, we have analysed the genomes of 20 healthy and wild-born chimpanzees and 5 healthy and wild-born bonobos (Table 1)^20^. These individuals were used to determine the geographical origin of Nico and to eliminate mutations not associated with the disease.

**Table 1:** Name, species and subspecies of the 25 individuals.

Name	Species	Subspecies	Code
Akwaya-Jean	*Pan troglodytes*	*ellioti*	Nigeria-Cameroon 1
Damian	*Pan troglodytes*	*ellioti*	Nigeria-Cameroon 2
Julie	*Pan troglodytes*	*ellioti*	Nigeria-Cameroon 3
Koto	*Pan troglodytes*	*ellioti*	Nigeria-Cameroon 4
Taweh	*Pan troglodytes*	*ellioti*	Nigeria-Cameroon 4
Alfred	*Pan troglodytes*	*troglodytes*	Central 1
Lara	*Pan troglodytes*	*troglodytes*	Central 2
Brigitte	*Pan troglodytes*	*troglodytes*	Central 3
Vaillant	*Pan troglodytes*	*troglodytes*	Central 4
Doris	*Pan troglodytes*	*troglodytes*	Central 5
Jimmie	*Pan troglodytes*	*verus*	Western 1
SeppToni	*Pan troglodytes*	*verus*	Western 2
Linda	*Pan troglodytes*	*verus*	Western 3
Cindy	*Pan troglodytes*	*verus*	Western 4
Alice	*Pan troglodytes*	*verus*	Western 5
Bwambale	*Pan troglodytes*	*schweinfurthii*	Eastern 1
Kidongo	*Pan troglodytes*	*schweinfurthii*	Eastern 2
Washu	*Pan troglodytes*	*schweinfurthii*	Eastern 3
Cleo	*Pan troglodytes*	*schweinfurthii*	Eastern 4
Maya	*Pan troglodytes*	*schweinfurthii*	Eastern 5
Hortense	*Pan paniscus*	—	Bonobo 1
Dzeeta	*Pan paniscus*	—	Bonobo 2
Hermien	*Pan paniscus*	—	Bonobo 3
Desmond	*Pan paniscus*	—	Bonobo 4
Natalie	*Pan paniscus*	—	Bonobo 5

### Variant calling and filtering

We used BWA-MEM (version 0.7.10-r789) algorithm to map the WGS data of the 26 individuals to the human reference genome hg19 (UCSC)^21^. FreeBayes^22^ (version 0.9.20) was used to call variants.

Potential false positives were filtered out by mapping quality (QUAL > 30) and read depth (DP > 5) of every polymorphic genotype. We also removed genomic variants for all positions that were not mapped in one or more individuals, as well as those sites substantially deviating from Hardy-Weinberg equilibrium. We removed positions with an allele balance lower than 0.2 and higher than 0.8 in heterozygous calls to account for possible contamination. Finally, just mappable positions were kept.

### Geographical origin of Nico

Since we have 5 individuals of each *Pan troglodytes* subspecies and we have the geographical origin for them, we ran a principal component analysis (PCA) using PLINK^23^ (version 1.90p) in order to classify the possible origin of Nico. PCA was performed using genome-wide data of all the 21 chimpanzees. Principal components 1, 2 and 3 were used to plot and visualize the results.

### Runs of homozygosity (ROHs)

The complete genome of all the individuals was divided into 1 Mbp windows with 2 kbp sliding windows. This allowed us to calculate the number of heterozygous positions per kbp and to identify ROHs present in their genomes. We did not use any statistical threshold to address ROHs, since the visual inspection did not reveal a substantial percentage of ROH, being only one present in chr7 (13 Mbp).

### Candidate genes

SnpEff^24^ (version 4.2) and SnpSift^25^ (version 4.2) were used on the dataset to predict the potential deleterious effect of the genetic variants (SNPs and indels) in the genes. SnpSift dbNSFP^26^ was used to add SIFT, PolyPhen2-HDIV and PolyPhen2-HVAR predictions, apart from others like PROVEAN or MutationTaster. Other parameters of interest, such as GERP scores, were also included. Similar approaches were successfully used in previous searches for candidate genes^27,28^.

Genetic variants predicted to have a high or moderate effect by SnpEff were selected as possible candidate mutations. Our strict selection criteria only included variants predicted as damaging by SIFT and probably damaging by PolyPhen2-HDIV and PolyPhen2-HVAR, the highest score for those predictors. Genes carrying private mutations of Nico and meeting the aforementioned features were crossed with the OMIM database.

### Data availability

The datasets generated during and analysed during the current study are available in the European Nucleotide Archive repository under accession code PRJEB21589.

## Results

### Genome information of Nico

We sequenced the whole genome of Nico on an Illumina HiSeq X Ten sequencing platform to a mean coverage of 30X. We found 22,399,631 total variants in his genome. 1,730 were considered as having a high effect by SnpEff (stop gained, frameshift, etc.), 46,782 were considered as having a moderate effect by SnpEff (missense, inframe insertion, etc.) and 83,855 were considered as having a low effect by SnpEff (synonymous, start retained, etc.). The rest of them were non-coding variants.

### Geographical origin of Nico

**Figure 2:**
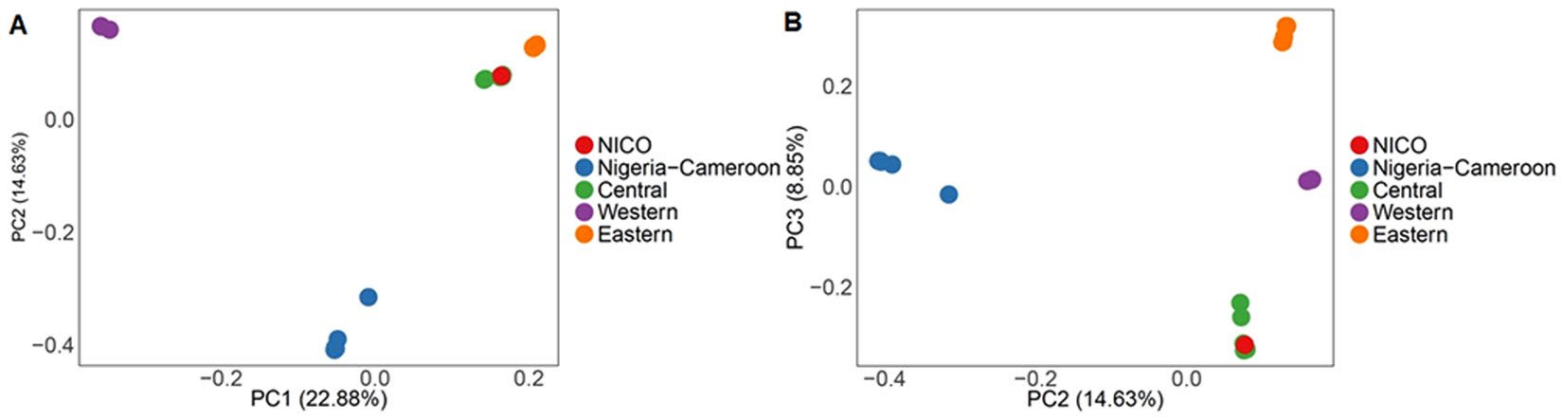
PCA. (**A**) PC1 (22.88% of variance) in x-axis and PC2 (14.63%) in y-axis. (**B**) PC2 in x-axis and PC3 (8.85%) in y-axis.

The variant calls for the complete genome were assessed for all the chimpanzees (Materials and methods). After PCA characterization, Nico clustered with central chimpanzees (Fig. 2).

PC1, which explains 22.88% of the variance, separates western from eastern and central chimpanzees. PC2, which explains 14.63% of the variance, separates Nigeria-Cameroon from the rest of chimpanzees^29^. Finally, PC3, which explains 8.85% of the variance, allows us to differentiate central from eastern chimpanzees.

### Runs of homozygosity

Runs of homozygosity are long sections of homozygous SNPs derived from consanguinity^30^ and they are commonly used to identify inbreeding. Therefore, the presence of ROHs in our studied individuals could indicate inbreeding into wild and accumulation of recessive alleles leading to recessive phenotypes.

**Figure 3:**
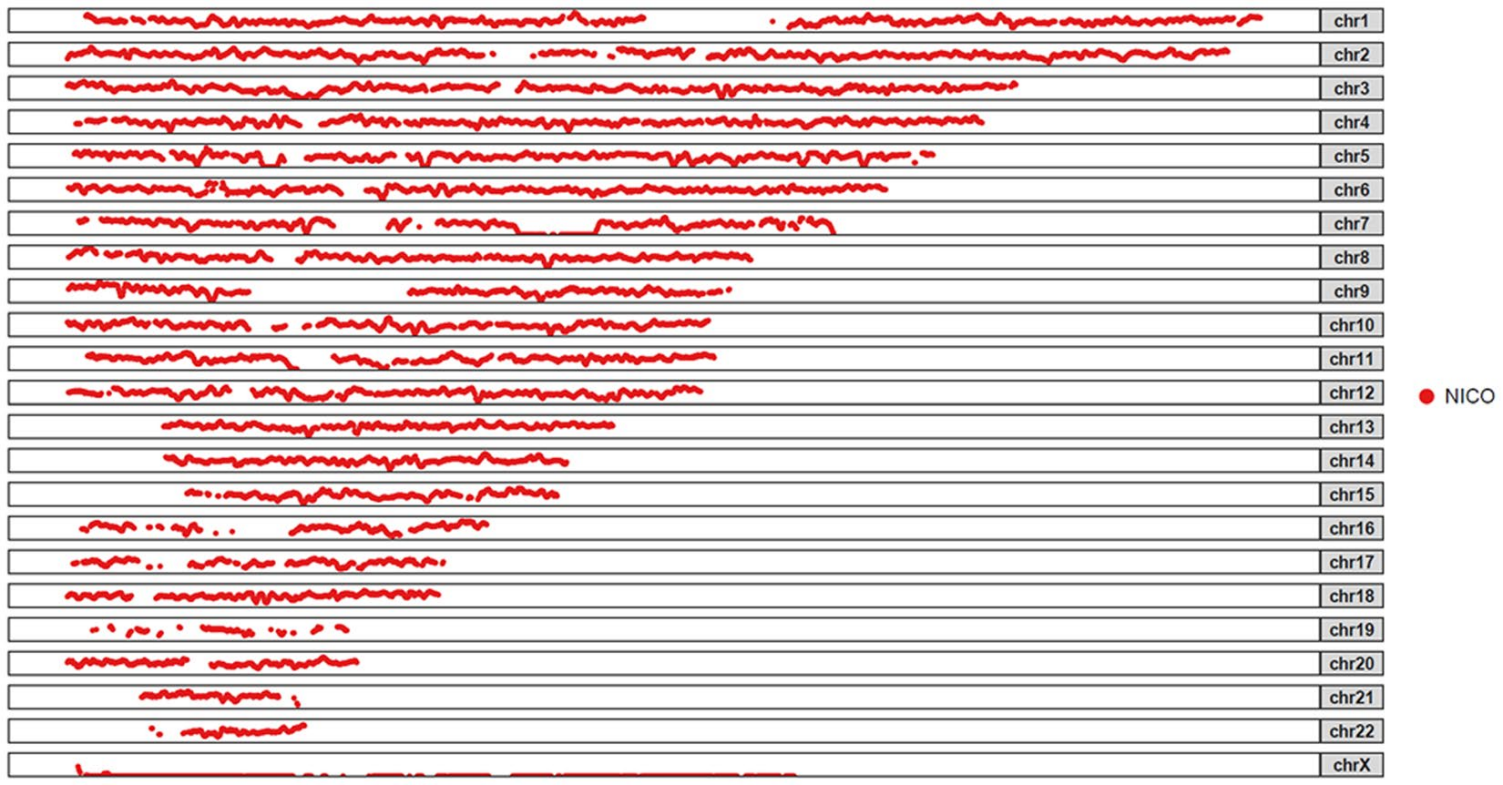
Number of heterozygous positions across the genome per kbp and per chromosome in Nico. The y-axis shows the heterozygosity and goes from 0 to 3 for each chromosome, while the x-axis shows the ordered positions for each chromosome.

Finding evidence of strong inbreeding in wild-born animals is improbable due to inbreeding avoidance mechanisms^31,32^, however it was worthwhile to discard it as a causal factor of Nico’s Chiari malformation. While considerable ROHs, and therefore measurable effects of inbreeding, explained the phenotype of the famous wild albino Western lowland gorilla, Snowflake^33^, no ROHs were detected in Nico (Fig. 3). Nico heterozygosity genome-wide was addressed and compared with the other central chimpanzees (Supplementary Figure S14) to give support to his not inbred origin theory.

### Candidate genes

When comparing the genomic diversity landscape of Nico to the rest of individuals, we identified 89 private mutations of high effect and 1,819 private mutations of moderate effect according to SnpEff. From the 89 mutations of high effect, 2 were considered as damaging by SIFT and as probably damaging by PolyPhen2-HDIV and PolyPhen2-HVAR and just one of them had an associated phenotype in OMIM. As this phenotype has an autosomal recessive inheritance and was found in Nico as heterozygous, we discarded all the high effect mutations. From the 1,819 moderate effect mutations, 184 were considered as damaging by SIFT and as probably damaging by PolyPhen2-HDIV and PolyPhen2-HVAR and 59 (in Supplementary Table S1) had an associated phenotype in OMIM. By considering just dominant phenotypes for heterozygous mutations, we removed 40 variants and kept 19 candidates. We also removed those phenotypes that only included the following OMIM categories: “nondiseases”, “susceptibility to multifactorial disorders” and “the relationship between the phenotype and gene is provisional”. We got a list of 15 candidates in which one gene appeared to be linked to bone density impairment pathologies: *LRP5*.

**Figure 4:**
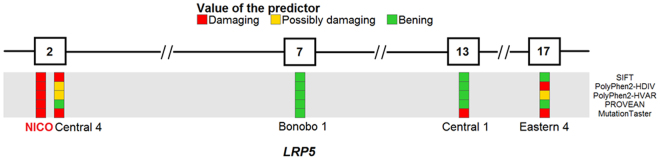
Schematic and zoomed representation of exons 2–17 of the *LRP5* gene along with all the mutations having a score for the used predictors. Each box is a predictor, from top to bottom: SIFT, PolyPhen2-HDIV, PolyPhen2-HVAR, PROVEAN and MutationTaster. The five mutations are missense variants in heterozygosity. The rest of the mutations that do not appear here are all of them considered as low by SnpEff, being most of them synonymous variants. For a full view of the mutations in *LRP5* in all the individuals, see Supplementary Fig. S17. Note: the mutation of the Central 4 have been moved to the right to make the visualization more comprehensive.

We found a private heterozygous mutation in Nico in *LRP5* gene (HGNC Approved Gene Symbol) (Fig. 4), in the position 68115354 of the chromosome 11 (hg19 coordinates). This gene, located in 11q13.2, encodes for the low-density lipoprotein receptor-related protein 5 (LRP5) and it contains 23 coding exons^34^ and spans 160 kbp^35^.

Nico presents a missense variant in one of his alleles of the *LRP5* gene. A transversion that changes a guanine for a thymine occurs in the second exon of the gene, in the position 131 of the coding sequence (out of 4,848). This causes a change of the original arginine for a leucine in the resultant protein in the position 44 (out of 1,615): c.131 G > T|p.Arg44Leu. This mutation happens in the extracellular domain, into the first β-propeller module (out of 4). It is predicted to have a moderate effect according to SnpEff and it is predicted as damaging according to SIFT and as probably damaging according to PolyPhen2-HDIV and PolyPhen2-HVAR (most harmful score for those predictors). Besides, other predictors as PROVEAN, MutationTaster or FATHMM consider it deleterious, disease causing and damaging respectively. The GERP (Genomic Evolutionary Rate Profiling) score, measured as rejected substitutions (or simply RS score), is 4.23. Previous studies employed a RS > 4 threshold to find deleterious genetic variants^36,37^. Actually, a study with 6,503 participants found that all genetic variants classified as pathogenic had a GERP > 2.95^38^.

A search in OMIM database of this gene reveals different human pathologies. Since the mutation is heterozygous, we were interested in those with an autosomal dominant (AD) inheritance pattern (Table 2).

**Table 2:** Phenotypes associated to mutations in *LRP5* gene. According to OMIM’s code, brackets indicate “nondiseases”, while braces indicate “susceptibility to multifactorial disorders”.

Phenotype	Phenotype MIM number	Inheritance
Hyperostosis, endosteal	144750	AD
Osteopetrosis, autosomal dominant	607634	AD
Osteosclerosis	144750	AD
van Buchem disease, type	607636	AD
[Bone mineral density variability]	601884	AD
{Osteoporosis}	166710	AD

We found several phenotypes with altered bone structure and bone density that could cause the Chiari malformation that Nico presents. Moreover, as previously mentioned, familial osteosclerosis has already been related to this malformation^19^.

## Discussion

Since they are our closest living relatives, non-human primates have been and are a very powerful source of information of our own species physiopathology. The advantages of non-human primate models are many due to the resemblances between them and us in several fields: genetics, immunology, behaviour, cognition, etc.^39^. However, although chimpanzees have been useful, for instance, in some viral diseases like hepatitis, where they are the classical reference^40,41^, ethical considerations, lack of individuals and long life cycle have made them a non-viable model for invasive studies in biomedical research.

More recently, the opinion toward the use of chimpanzees in research have become critical and reluctant arguing ethical reasons and their poor contribution to the progress in the biomedical field^42,43^. On the other hand, other authors suggest that the policy carried out in the US by organisms like the National Institutes of Health (NIH) and the United States Fish and Wildlife Services (USFWS) over the last lustrum has impeded that progress and has stopped valuable scientific research^44^.

In the middle of this debate, we demonstrated in the present research that, instead of using the animals to induce them diseases, we can study their natural conditions. On top of that, this can be an attractive scenario to study the diseases from an evolutionary perspective.

Here, we presented the first case of a chimpanzee diagnosed with the Chiari malformation and suggested its genetic background. Finding specimens with similar traits to the ones presented by humans can be an excellent tool to acquire new knowledge of the physiopathology of different diseases and can help, eventually, fighting against them. It will be interesting, in the light of biomedical research improvement, to study animals with different pathologies if we have the opportunity. Furthermore if those animals are our closest living relatives, as they should in principle reproduce human pathologies better than other animal models.

We sequenced the whole genome of Nico, a central chimpanzee diagnosed with the Chiari malformation. We found no evidence of inbreeding in his genome, but we found a private, predicted as damaging mutation in *LRP5* gene that could explain his phenotype.

LRP5 protein has a great impact in Wnt signalling pathway. This pathway is highly conserved across species and it is crucial in several critical processes such as central nervous system development^45,46^ and body-axis formation^47^. As for what concern us, Wnt signalling pathway also controls bone density and bone metabolism, which affects osteoblast growth and differentiation^48^. LRP5 is a membrane co-receptor for the secreted protein Wnt, being the receptor the protein Frizzled. The interaction of these three proteins activates the canonical Wnt signalling pathway. This allows β-catenin to interact with different transcription factors that modify the expression of some important genes in osteoblasts^49^. This way, *LRP5* gene plays a key role in bone homeostasis and several skeletal pathologies, such as osteoporosis, are related to mutations in its coding region^50^. Different mutations in *LRP5* have been linked to reductions in bone mineral density, what could confer the individuals that present one of them susceptibility to the aforementioned osteoporosis^51^. On the other hand, activating mutations in this gene cause an increase of bone mineral density^52^. These facts demonstrate the great importance of *LRP5* in bone.

Our findings are supported by a previously reported case linking *LRP5* and a Chiari malformation phenotype^53^. In it, a different missense mutation than the one found in Nico was identified, although it affected the same exon of the gene and the same β-propeller domain of the protein. This is the p.Gly171Val mutation, described prior to the other publication^54^. Besides, some other missense variants affecting as well the first β-propeller domain have been reported to cause a high bone density phenotype with an autosomal dominant inheritance^55^.

These mutations are supposed to cause a gain of function. Although it is not clear, they presumably affect the normal, physiological inhibition of the Wnt pathway defined above by not allowing a proper interaction of LRP5 and the protein DKK1, one of its inhibitors^56^.

Further explorations are needed despite our findings. In the first place, it will be necessary to confirm the mutation of Nico in *LRP5* gene. Then, it must be proved that it is affecting the function of the protein. Finally, it will be interesting to study a cohort of humans presenting the Chiari malformation to try to find the same or other different mutations in *LRP5* gene, considering that the evidences connecting both are little. This will mean that alterations in the function of LRP5 can be somehow implicated in the pathophysiology of the malformation.

## Conclusions

We found a novel predicted as damaging mutation in *LRP5* gene in Nico, a chimpanzee with the Chiari malformation. We suggest, along with previous results, a genotype-phenotype association between the gene and the pathology, both in chimpanzees and humans. This would be the first example in which the chimpanzee condition and genetic background provided insights of a human medical condition.

A single gene mutated may not be the unique explanation to the complex phenotype of Nico. It is likely that other genes, regulatory elements and environmental factors are implicated. However, this study can be a valuable starting point for new research, and it may provide novel and important knowledge to try to comprehend the same pathology in humans.

## Electronic supplementary material

Supplementary_information

Supplementary Table 1
