# Author Correction: Potential damaging mutation in *LRP5* from genome sequencing of the first reported chimpanzee with the Chiari malformation

**DOI:** 10.1038/s41598-018-24993-w

**Published:** 2018-05-04

**Authors:** Manuel Solis-Moruno, Marc de Manuel, Jessica Hernandez-Rodriguez, Claudia Fontsere, Alba Gomara-Castaño, Cristina Valsera-Naranjo, Dietmar Crailsheim, Arcadi Navarro, Miquel Llorente, Laura Riera, Olga Feliu-Olleta, Tomas Marques-Bonet

**Affiliations:** 1 Institut de Biologia Evolutiva (CSIC-UPF), Departament de Ciències Experimentals i de la Salut, Universitat Pompeu Fabra, Doctor Aiguader 88, Barcelona, 08003 Spain; 2 Fundació Mona, Carretera C-25, s/n, 17457 Riudellots de la Selva, Girona, Spain; 3 Catalan Institution of Research and Advanced Studies (ICREA), Passeig de Lluís Companys, 23, Barcelona, 08010 Spain; 4 CNAG-CRG, Centre for Genomic Regulation, Barcelona Institute of Science and Technology (BIST), Baldiri i Reixac 4, Barcelona, 08028 Spain

Correction to: *Scientific Reports* 10.1038/s41598-017-15544-w, published online 09 Novenber 2017

In the original version of this Article, Miguel Lorente, Laura Riera and Olga Feliu-Olleta were incorrectly affiliated with ‘Catalan Institution of Research and Advanced Studies (ICREA), Passeig de Lluís Companys, 23, 08010 Barcelona’. The correct affiliation is listed below.

Fundació Mona, Carretera C-25, s/n, 17457 Riudellots de la Selva, Girona, Spain

This error has now been corrected in the PDF and HTML versions of the Article and in the accompanying Supplementary Information file.

