# Supplementary material for: Potential damaging mutation in LRP5 from genome sequencing of the first reported chimpanzee with the Chiari malformation
Source: Sci Rep. 2017 Nov 9;7:15224. doi: 10.1038/s41598-017-15544-w (PMC5680330; doi:10.1038/s41598-017-15544-w)
Supplement: Supplementary file 1 — Supplementary_information [file 41598_2017_15544_MOESM1_ESM.pdf]

Potential damaging mutation in *LRP5* from genome sequencing of the first reported chimpanzee with the Chiari malformation

Manuel Solis-Moruno[1], Marc de Manuel[1], Jessica Hernandez-Rodriguez[1], Claudia Fontsere[1], Alba Gómara-Castaño[2], Cristina Valsera-Naranjo[2], Dietmar Crailsheim[2], Arcadi Navarro[1,3,4], Miquel Llorente[2], Laura Riera[2], Olga Feliu-Olleta[2], Tomas Marques-Bonet[1,3,4]

[1] *Institut de Biologia Evolutiva (CSIC-UPF), Departament de Ciències Experimentals i de la Salut, Universitat Pompeu Fabra, Doctor Aiguader 88, 08003 Barcelona, Spain.*

[2] *Fundació Mona, Carretera C-25, s/n, 17457 Riudellots de la Selva, Girona, Spain.*

[3] *Catalan Institution of Research and Advanced Studies (ICREA), Passeig de Lluís Companys, 23, 08010 Barcelona, Spain.*

[4] *CNAG-CRG, Centre for Genomic Regulation, Barcelona Institute of Science and Technology (BIST), Baldiri i Reixac 4, 08028 Barcelona, Spain.*

Supplementary Information

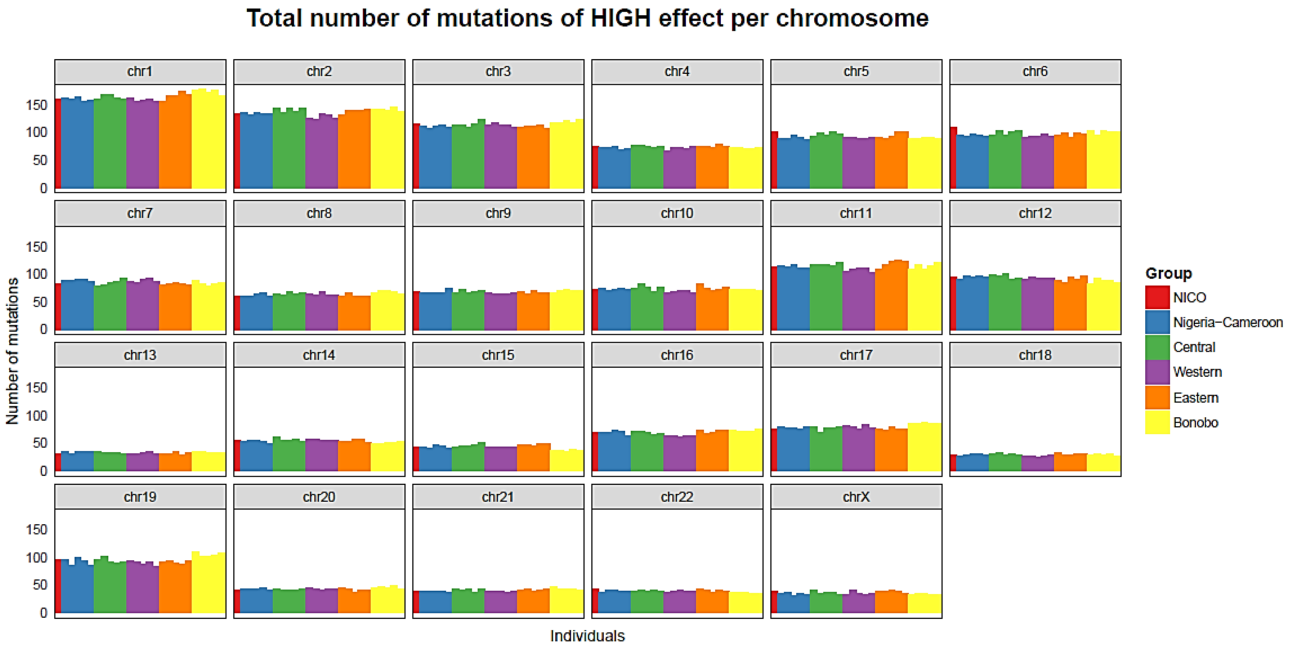

Supplementary Figure S1. Total number of mutations per chromosome of high effect according to SnpEff.

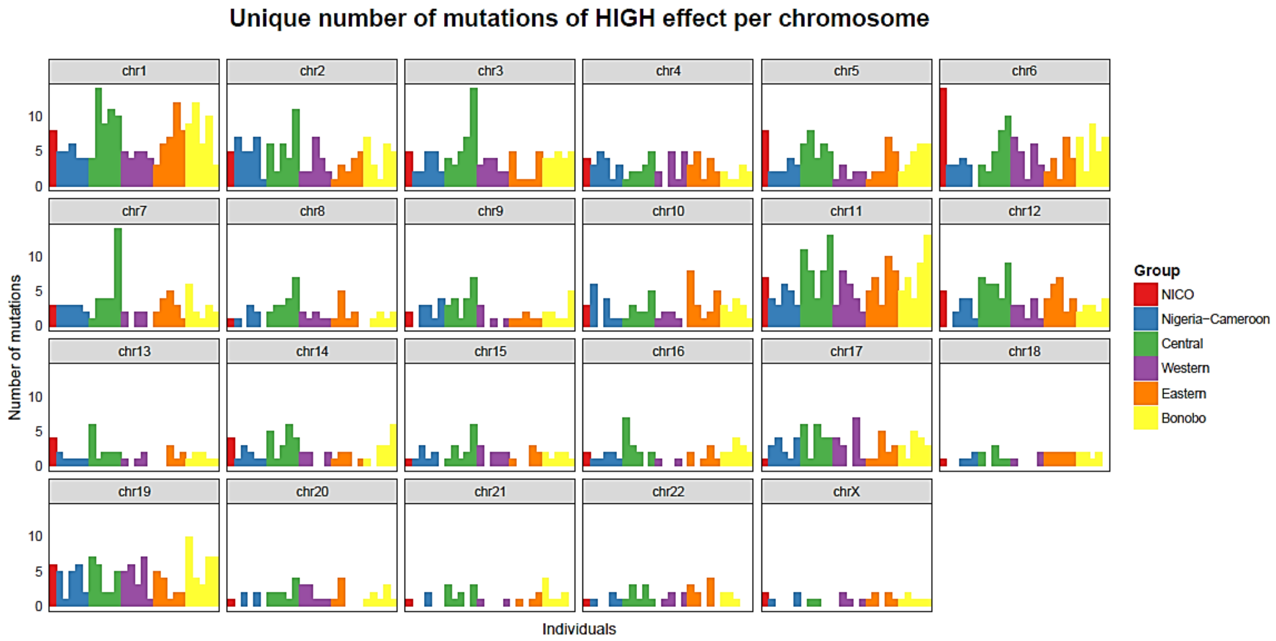

Supplementary Figure S2. Private mutations per chromosome of every individual of high effect according to SnpEff.

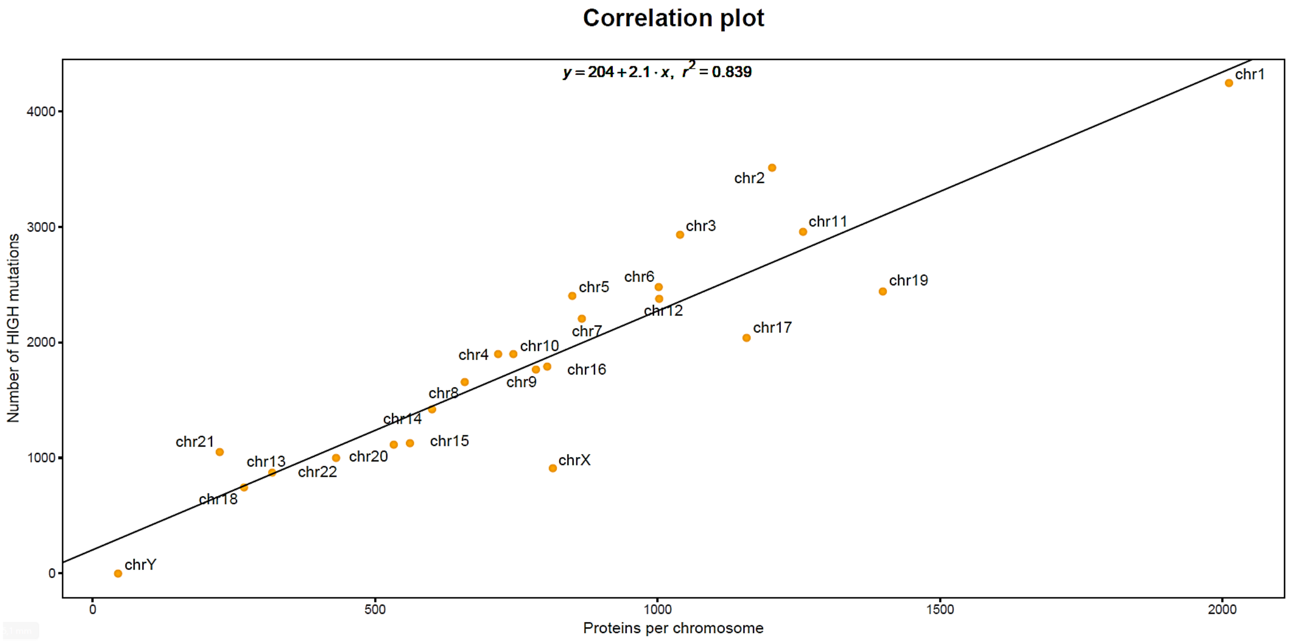

Supplementary Figure S3. Correlation plot showing the number of proteins encoded per chromosome and the number of high effect mutations according to SnpEff. We found that those numbers correlate quite well, with a $r^2 = 0.839$.

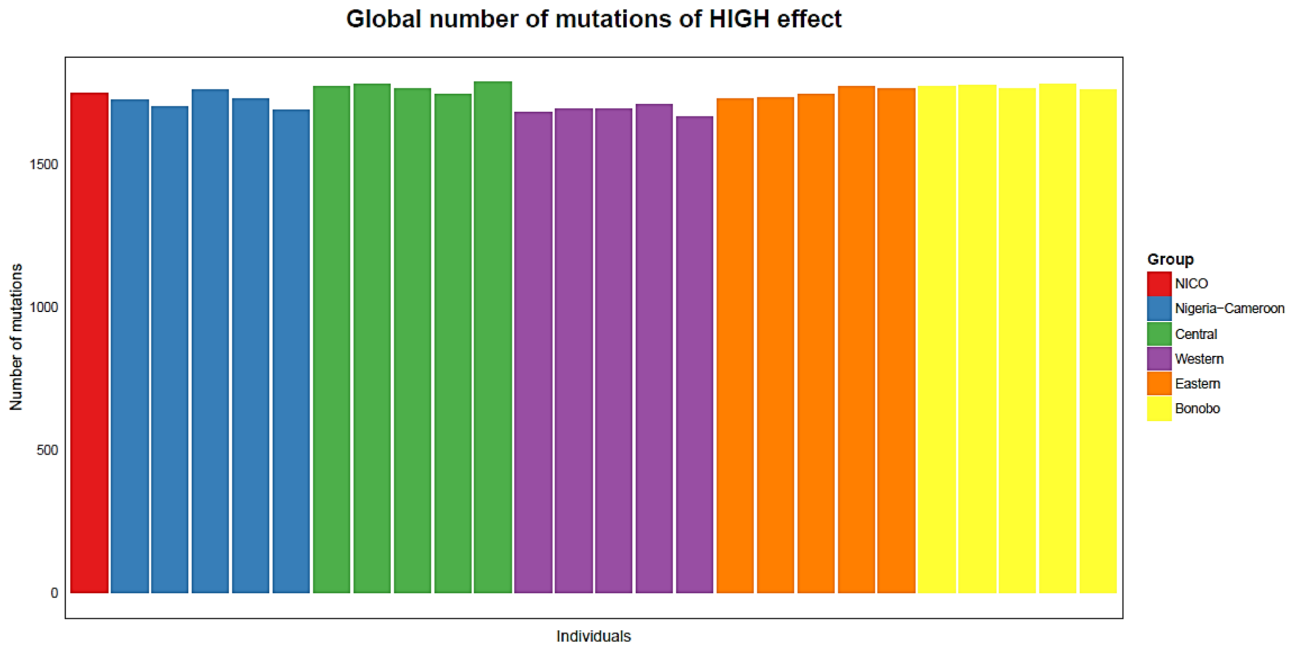

Supplementary Figure S4. Whole number of mutations of high effect according to SnpEff per individual.

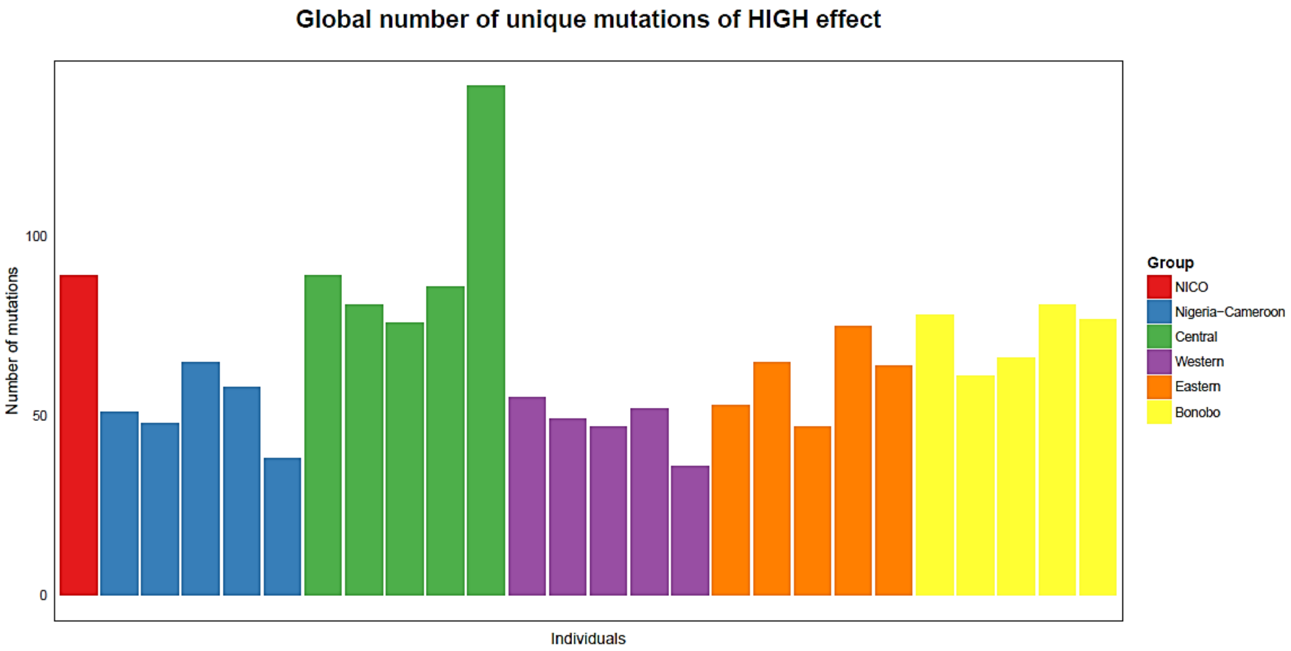

Supplementary Figure S5. Whole number of private mutations of high effect according to SnpEff per individual.

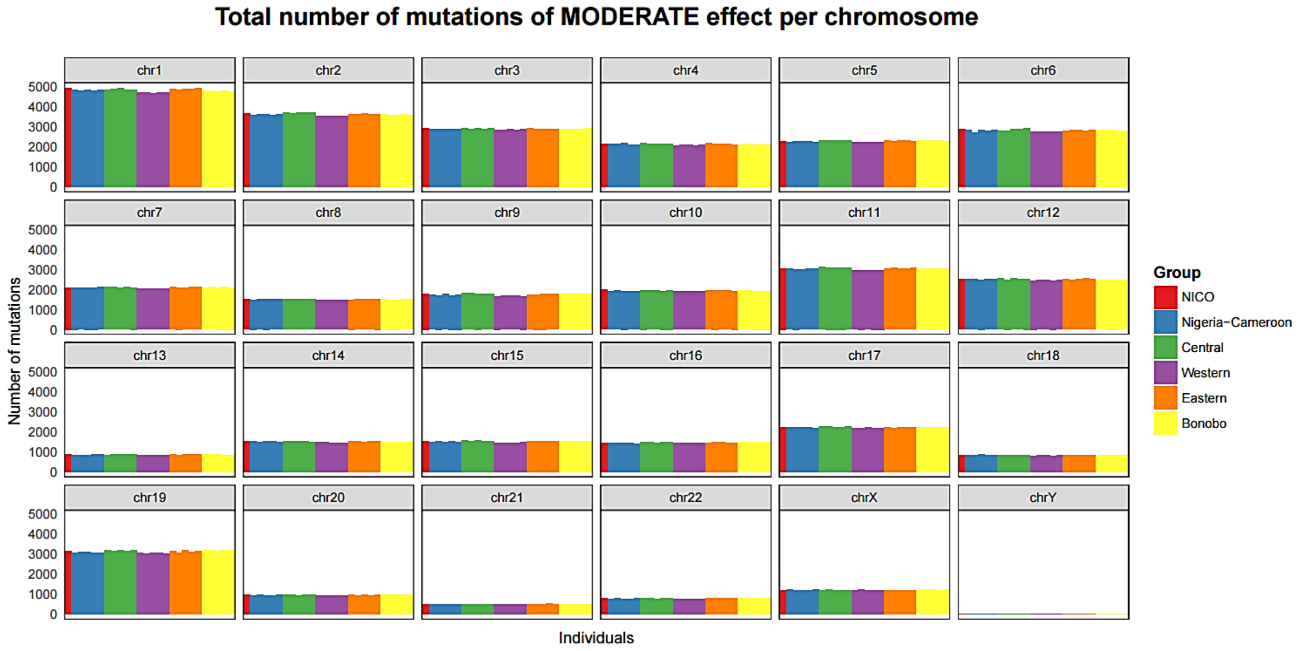

Supplementary Figure S6. Total number of mutations per chromosome of moderate effect according to SnpEff.

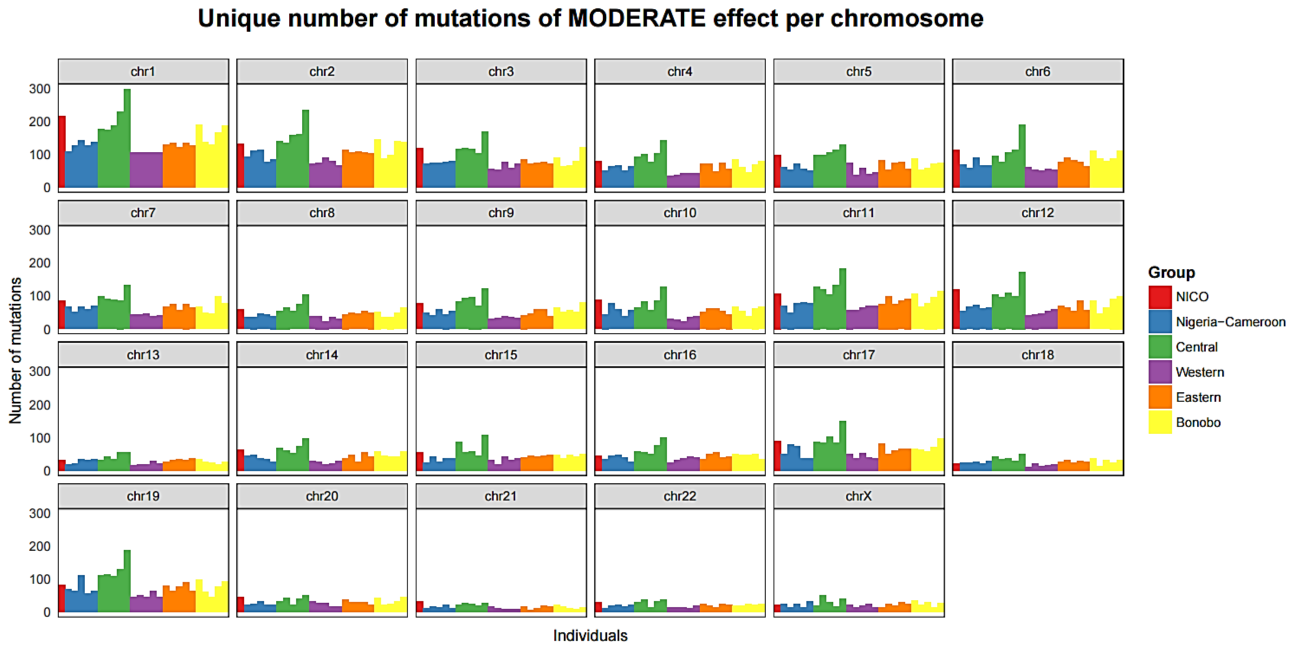

Supplementary Figure S7. Private mutations per chromosome of every individual of moderate effect according to SnpEff.

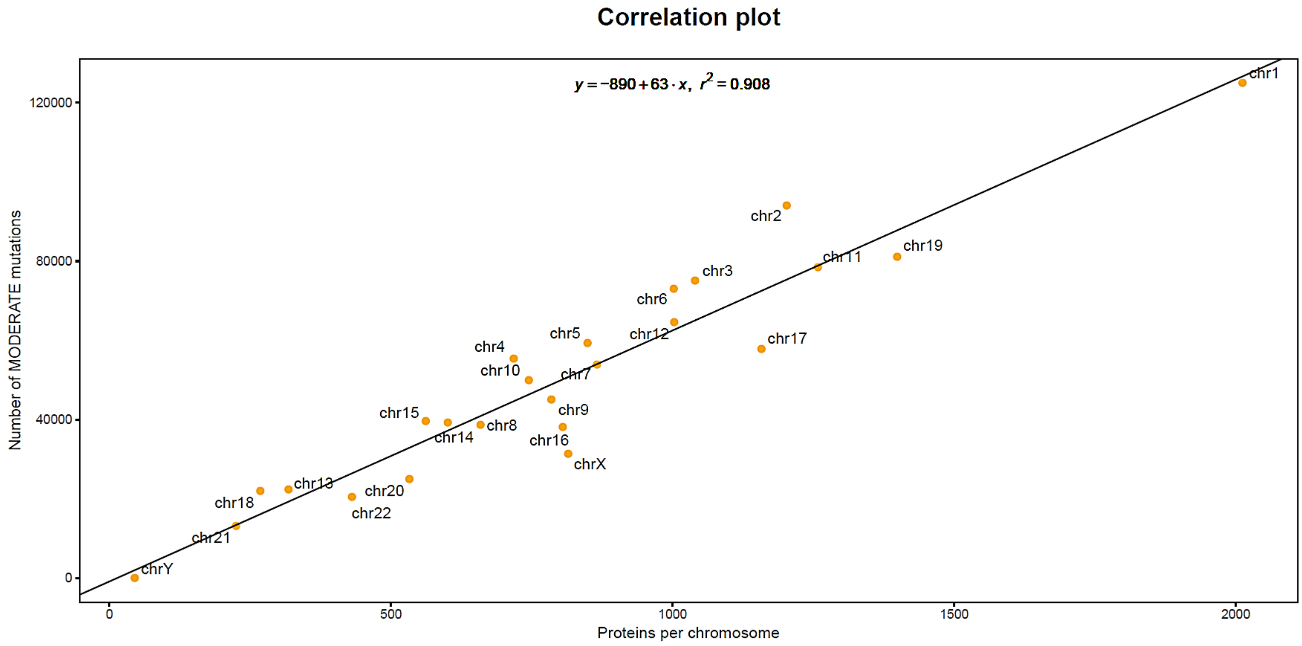

Supplementary Figure S8. Correlation plot showing the number of proteins encoded per chromosome and the number of moderate effect mutations according to SnpEff. We found that those numbers correlate even better than the one of high effect, with $r^2 = 0.908$.

What we see with the HIGH effect mutations and the MODERATE effect mutation is that there is higher number of mutations in chromosome 19 than in some others that are much larger. To prove that there were no errors in our analysis, we did a correlation with the number of proteins encoded per chromosome (extracted from ENSEMBL. Chr1: http://www.ensembl.org/Homo_sapiens/Location/Chromosome?r=1) and the number of mutations that we found in every one of them. The r^2 is 0.839 and 0.908 for high and moderate effect mutations respectively. Besides, it is known that chromosome 19 has the biggest gene density[1].

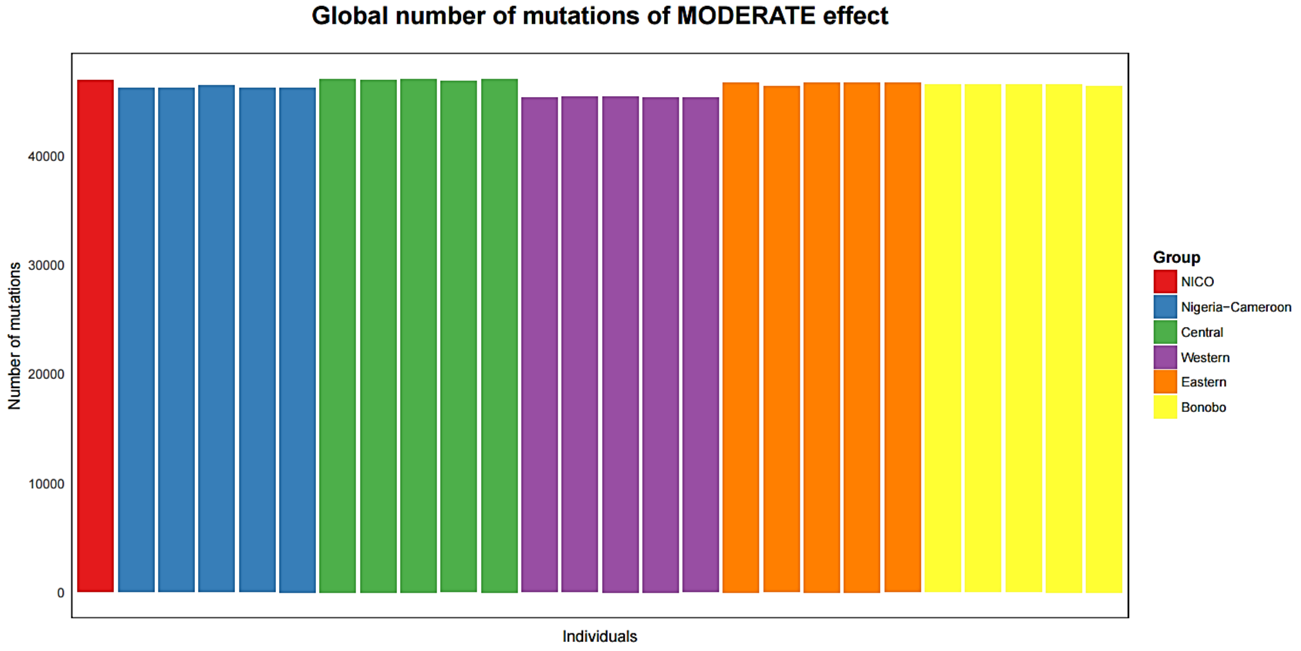

Supplementary Figure S9. Whole number of mutations of moderate effect according to SnpEff per individual.

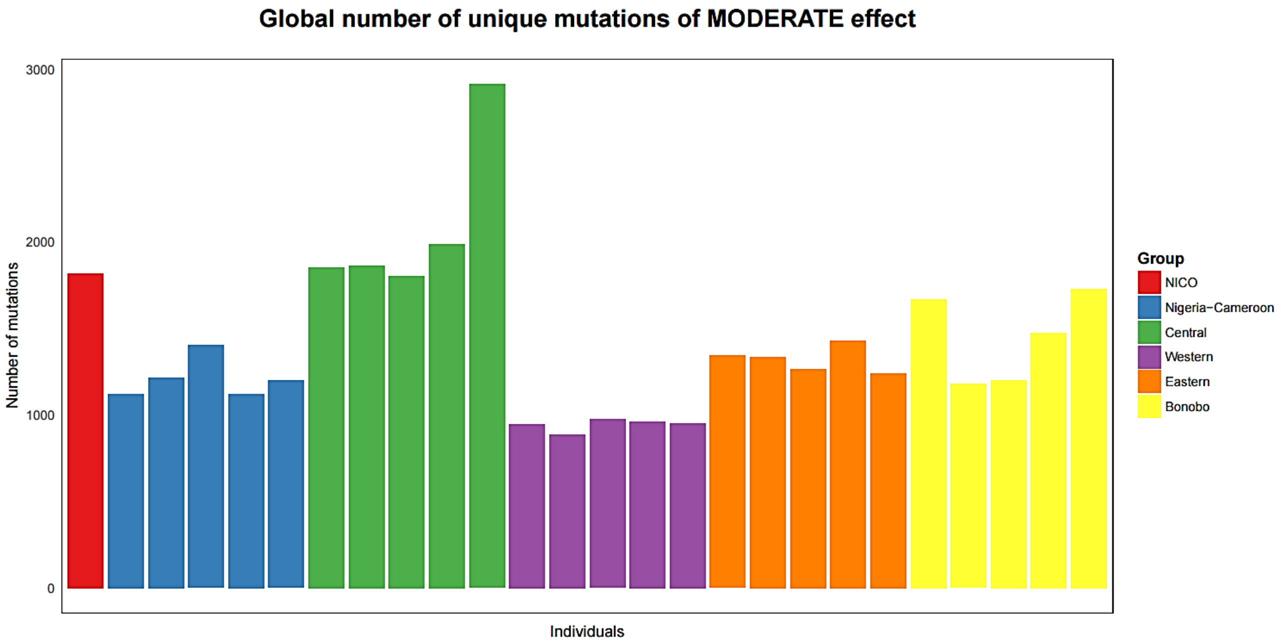

Supplementary Figure S10. Whole number of private mutations of moderate effect according to SnpEff per individual.

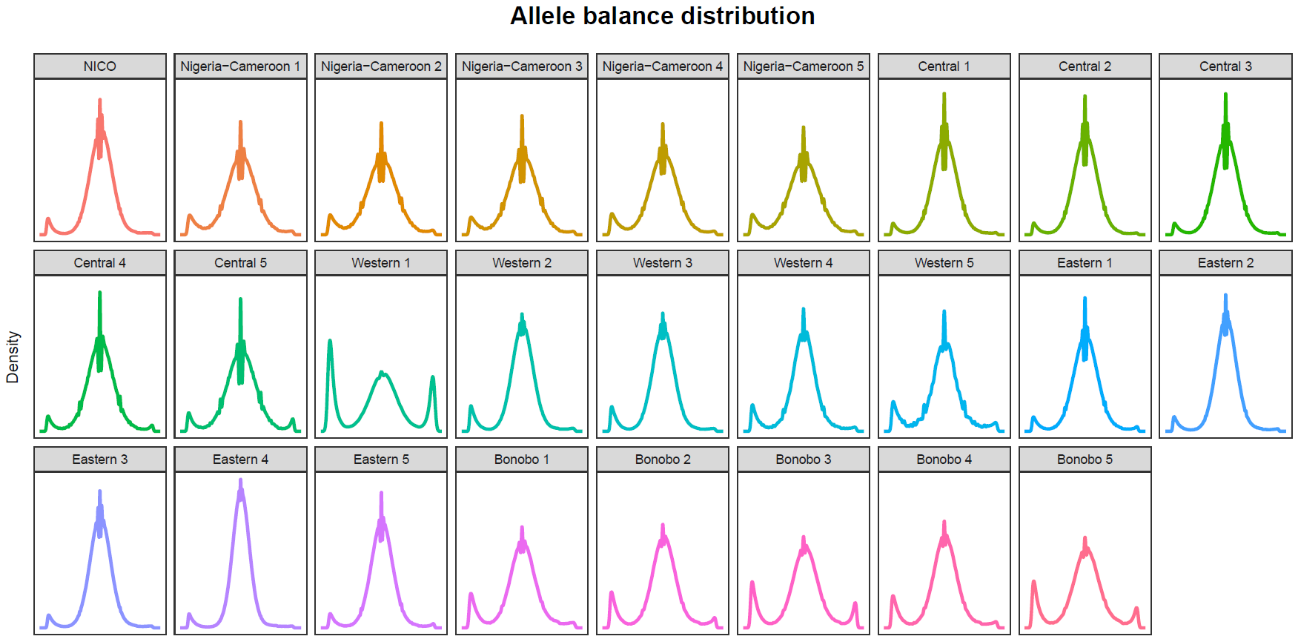

Supplementary Figure S11. Allele balance distribution per sample before the filtering. The skewed peaks could indicate contamination.

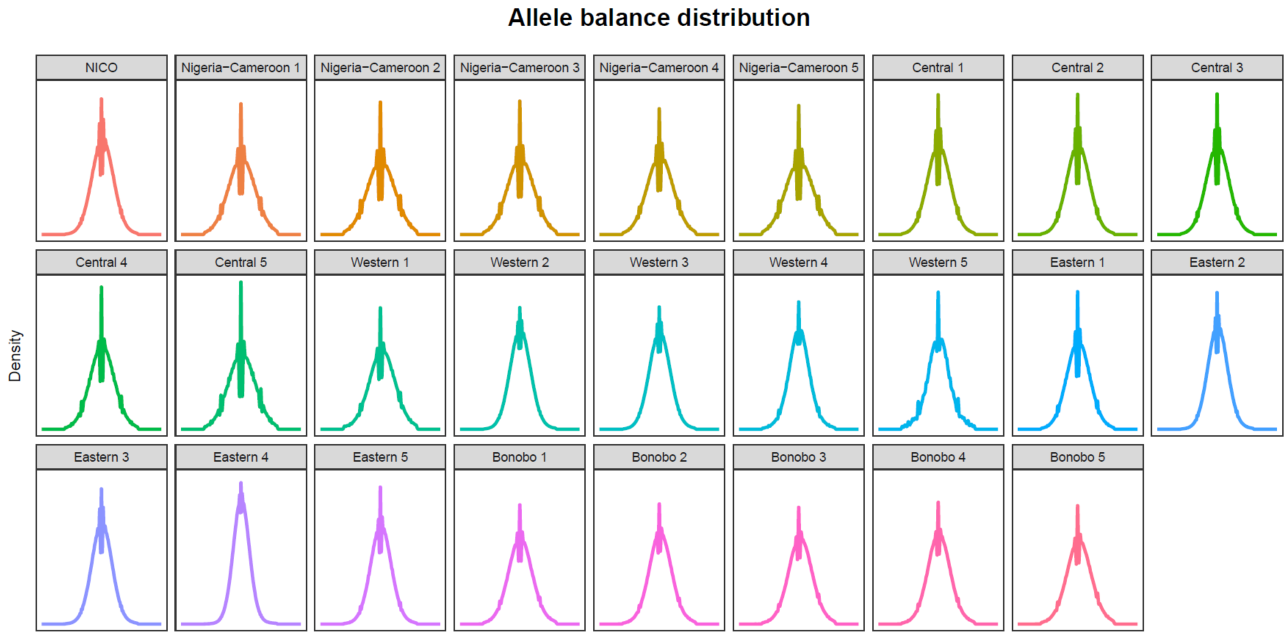

Supplementary Figure S12. Allele balance distribution per sample after the filtering. We observe how the peaks are considerably reduced in every sample.

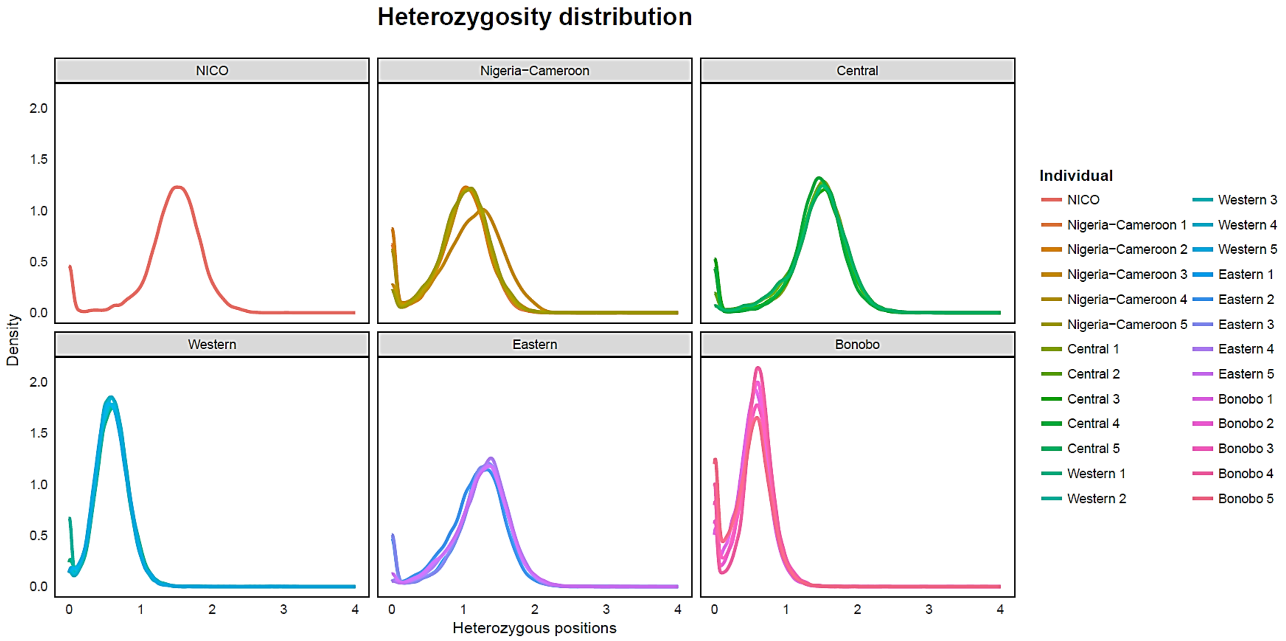

Supplementary Figure S13. Density plot showing the distribution of heterozygous positions of 1 Mbp windows with 2 kbp sliding windows of every individual by subspecies. It can be seen how they then to behave similarly.

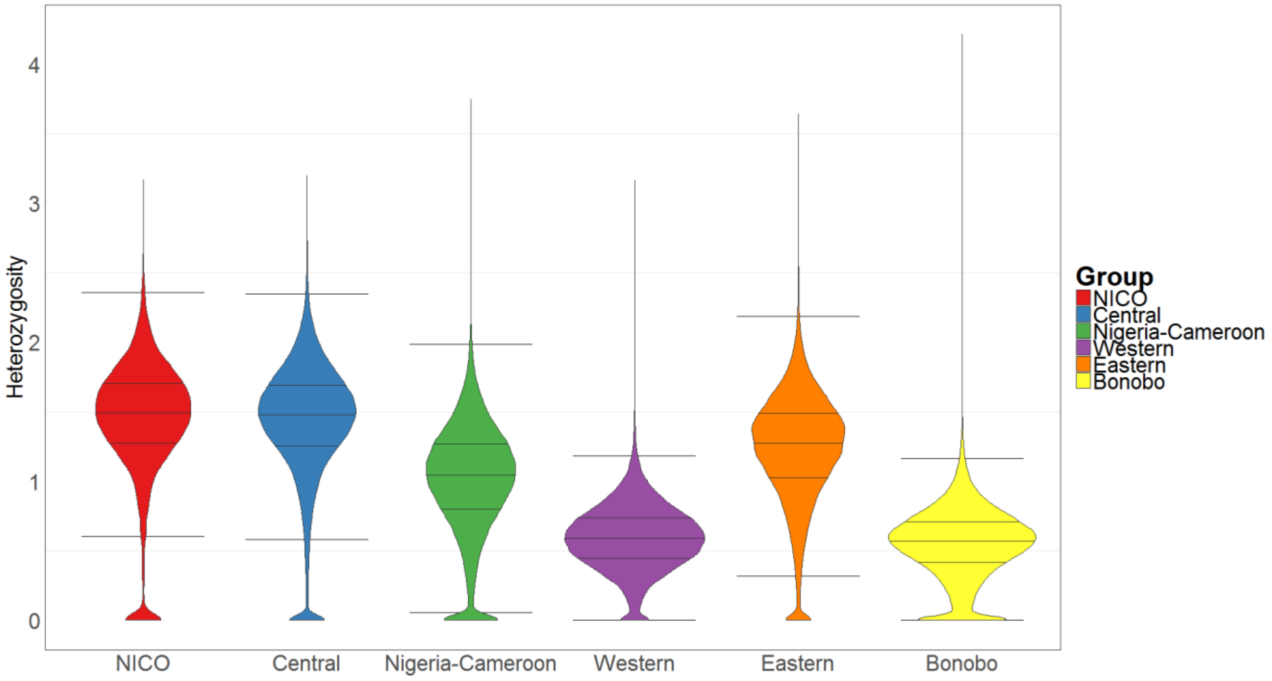

Supplementary Figure S14. Violin plot showing average genomic heterozygosity (in heterozygous positions per kbp). It can be seen how the heterozygosity of Nico behaves similarly to the group of the other central chimpanzees.

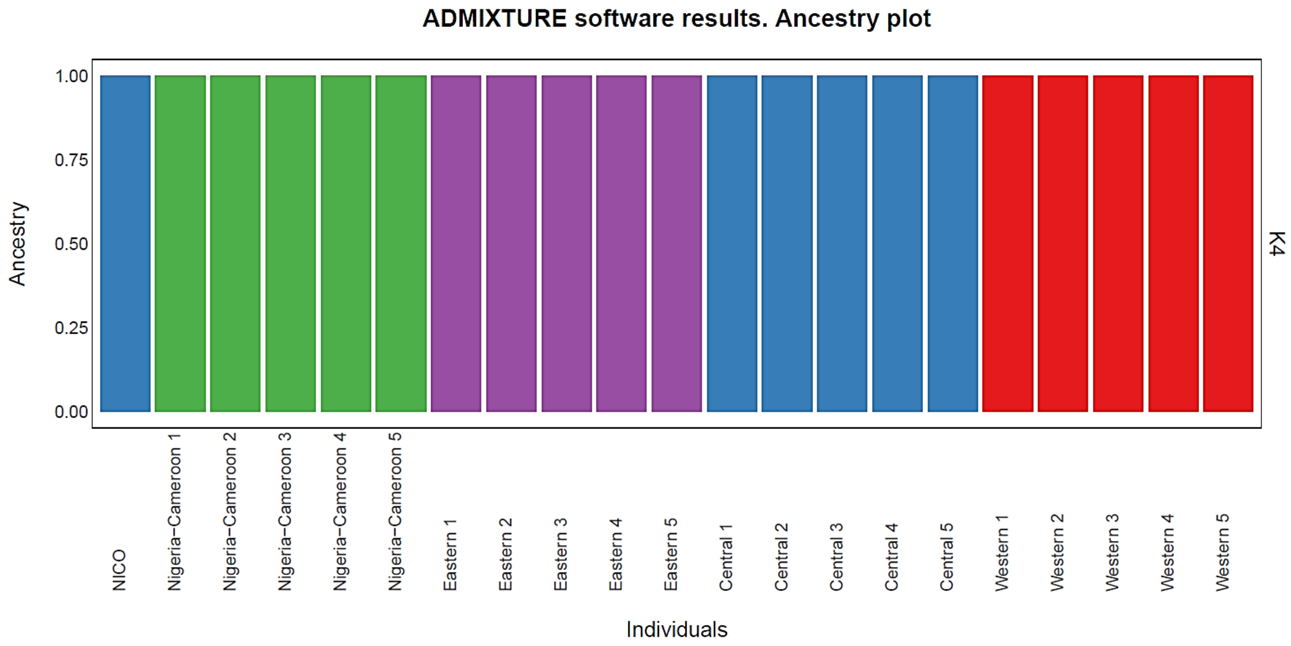

Supplementary Figure S15. ADMIXTURE software[2] was used to reinforce the results of the PCA. Here, we show the plot that explores the ancestry of Nico among the 4 chimpanzee subspecies (*K*=4). This result, along with the observed in the PCA, indicates non-admixture patterns in all of the individuals. We used just SNP data for this analysis, as well as with the PCA.

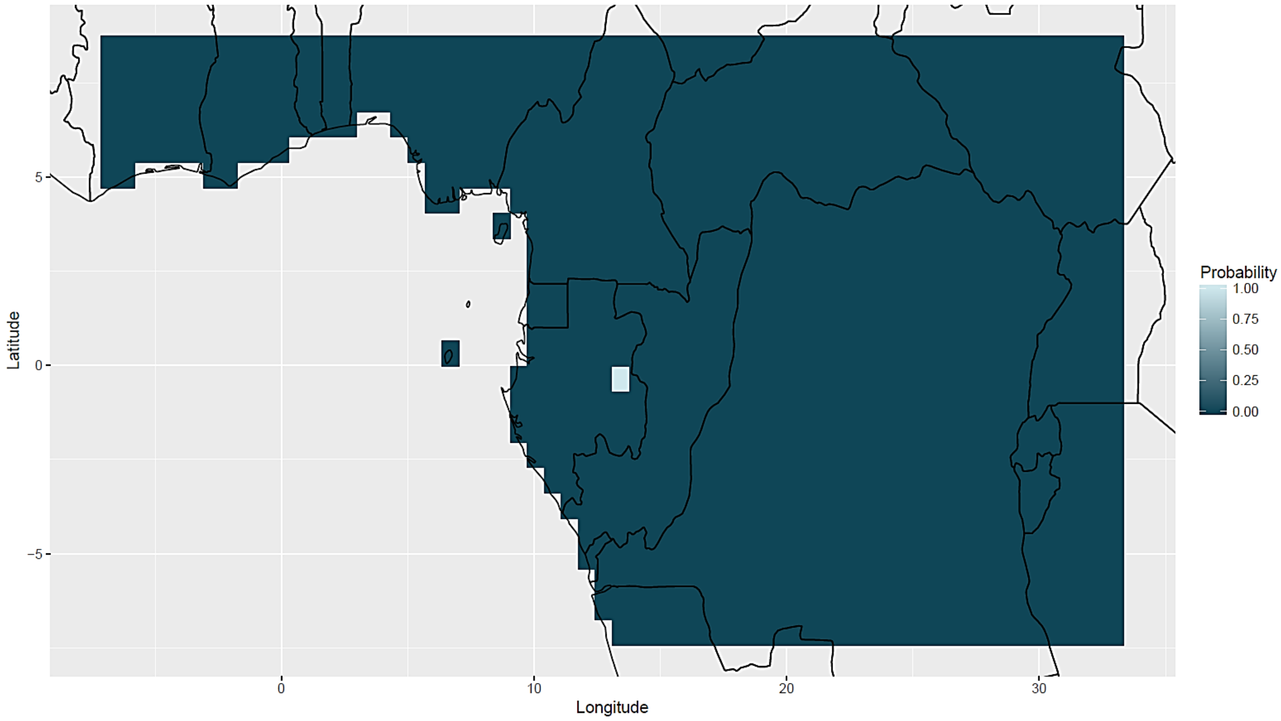

Supplementary Figure S16. Heatmap with the probabilities of Nico belonging to a particular region was generated using OriGen[3] (version 1.4.3) package of R software (https://cran.r-project.org/) (version 3.3.1). We used a non-admixed assumption (results from ADMIXTURE software and PCA).

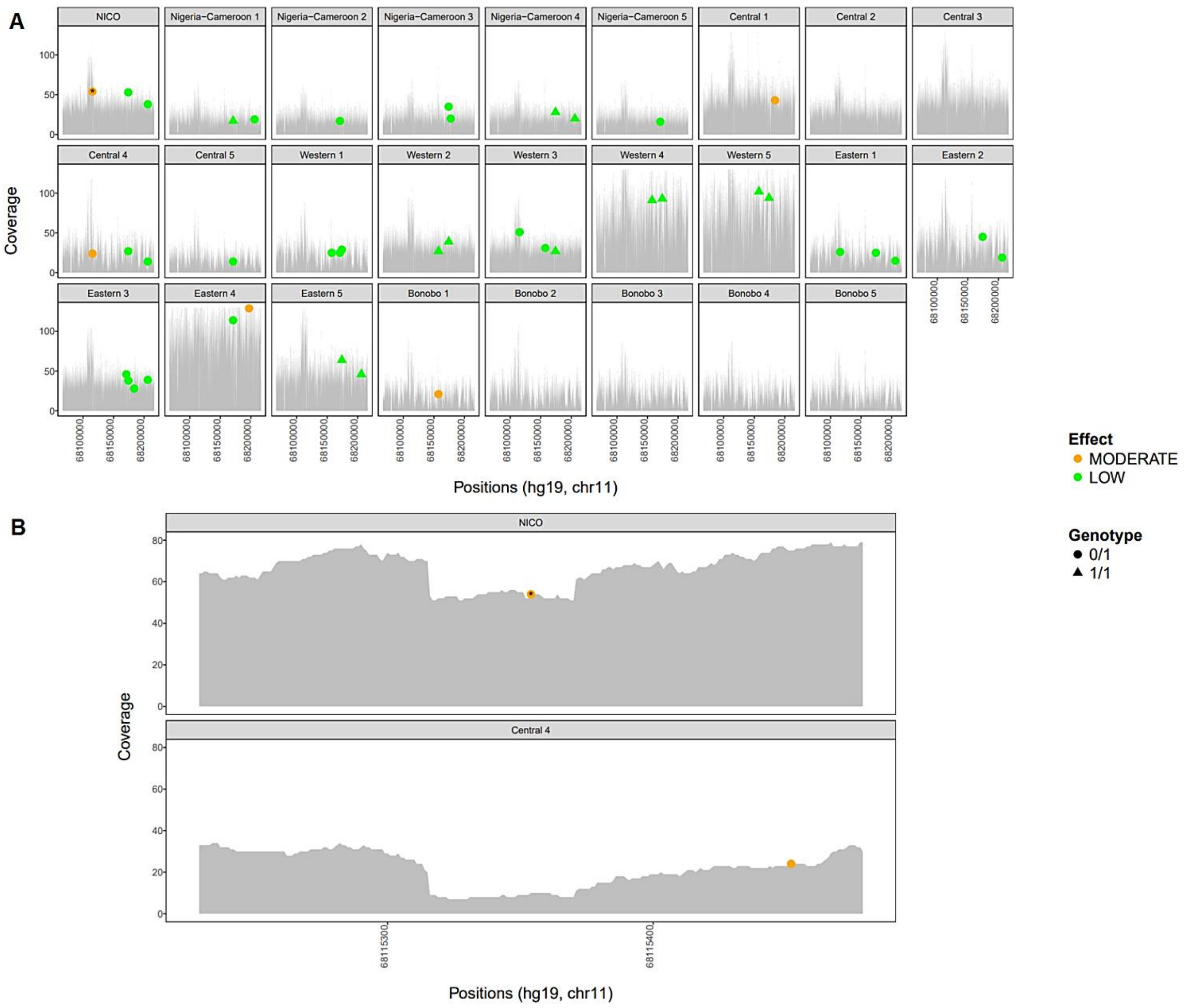

Supplementary Figure S17. *LRP5*. **A.** Coverage and genotype per individual for all the variants found in the whole *LRP5* gene. **B.** Zoomed region showing coverage and genotype in Nico and in Central 4 for the 250 bp surrounding the mutation of interest. Fixed mutations in the 26 individuals are not shown. This image complements the one of the main text. 0/1 indicates heterozygous genotype and 1/1 homozygous genotype. The shown effect is according to SnpEff. Note that there are some moderate effect mutations (all of them missense variants in heterozygosity), but only the one present in Nico (mark with asterisk) is classified with the highest score for SIFT, PolyPhen2-HDIV and PolyPhen2-HVAR. The genomic coordinates of *LRP5* gene were extracted from the NCBI and converted from hg38 to hg19 using UCSC's LiftOver.

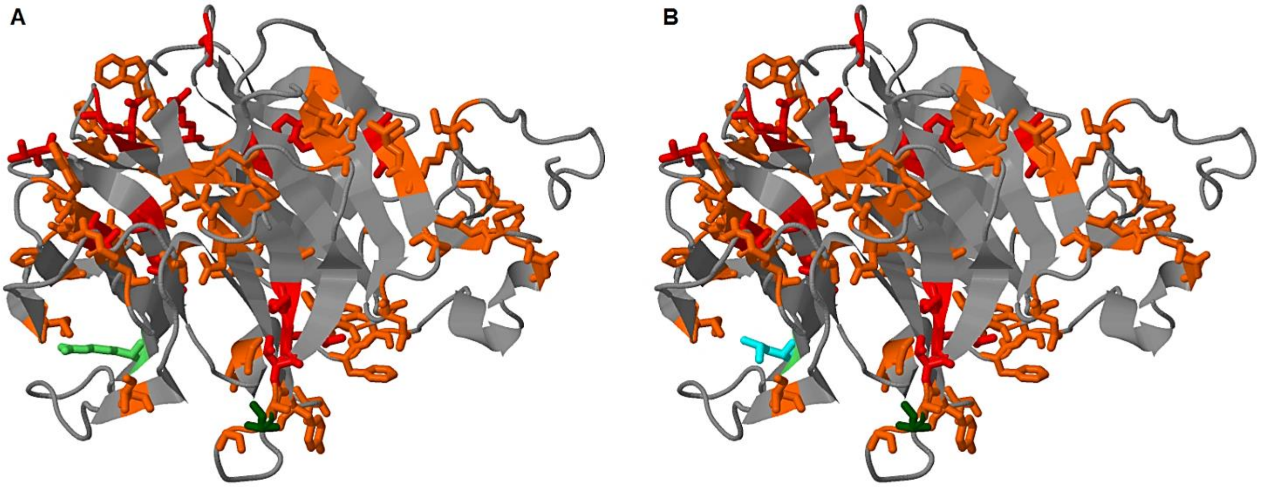

Supplementary Figure S18. G23D screenshots for the domain of LRP5 in which the mutation of interest is located. **A.** Wildtype domain, with the arginine in green. **B.** Mutated domain, with the leucine in blue.

Supplementary Bibliography.

1. Grimwood, J. *et al.* The DNA sequence and biology of human chromosome 19. *Nature* **428,** 529–535 (2004).

2. Alexander, D. H., Novembre, J. & Lange, K. Fast model-based estimation of ancestry in unrelated individuals. *Genome Res.* **19,** 1655–1664 (2009).

3. Rañola, J. M., Novembre, J. & Lange, K. Fast spatial ancestry via flexible allele frequency surfaces. *Bioinformatics* **30,** 2915–2922 (2014).
